# Supplementary material for: The post-abscission midbody is an intracellular signaling organelle that regulates cell proliferation
Source: Nat Commun. 2019 Jul 18;10:3181. doi: 10.1038/s41467-019-10871-0 (PMC6639393; doi:10.1038/s41467-019-10871-0)
Supplement: Supplementary file 2 — Description of Additional Supplementary Files [file 41467_2019_10871_MOESM2_ESM.pdf]

Description of Additional Supplementary Files

File Name: Supplementary Data 1
Description: mRNAs up-regulated and down-regulated in cells enriched for post-mitotic MBs. The results shown are derived and analyzed from RNAseq analysis of three independent libraries for every experimental condition (GFP- and GFP+). Each library was generated from independent flow sorting experiment.

File Name: Supplementary Data 2
Description: Purified post-abscission MB proteome
Red marks matricellular receptors identified in proteomic analysis. Gray labels proteins that previously have been shown to be present in MBs. Yellow marks putative contaminants.

File Name: Supplementary Data 3
Description: Primers used for qPCR analysis

File Name: Supplementary Data 4
Description: mRNAs up-regulated/down-regulated in cells containing internalized post-abscission GFP-MBs. HeLa cells were incubated with purified GFP-MBs for three hours. Cells were then separated in pools with or without internalized GFP-MBs using flow cytometry. Both pools were then processed for RNAseq analysis. The data shown is generated from four different GFP-MB positive and negative pools of cells. The only mRNAs below FDR of 0.01 is shown in the table.

File Name: Supplementary Data 5
Description: mRNAs up-regulated in cells containing internalized post-abscission GFP-MBs are known to induce proliferation.
(A) Pathway analysis of mRNAs upregulated in cells containing internalized GFP-MBs. Only mRNAs with FDR values lower then 0.05 were included in this analysis.
(B) The extend of upregulation of mRNAs that were also identified and validated in first RNAseq analysis.

File Name: Supplementary Movie 1
Description: Tomography tilt series of post-abscission MB being internalized by HeLa cell.

File Name: Supplementary Movie 2
Description: 3D rendering of the model based on tomography tilt series shown in Supplemental Movie 1.

File Name: Supplementary Movie 3
Description: The mobility of GFP-MB taken up by cell expressing mCherry-LifeAct
