# Supplementary material for: The post-abscission midbody is an intracellular signaling organelle that regulates cell proliferation
Source: Nat Commun. 2019 Jul 18;10:3181. doi: 10.1038/s41467-019-10871-0 (PMC6639393; doi:10.1038/s41467-019-10871-0)
Supplement: Supplementary file 11 — Supplementary_info_NEW [file 41467_2019_10871_MOESM11_ESM.pdf]

# The Post-Abscission Midbody is an Intracellular Signaling Organelle that Regulates Cell Proliferation

Peterman et al

Supplementary Figure 1

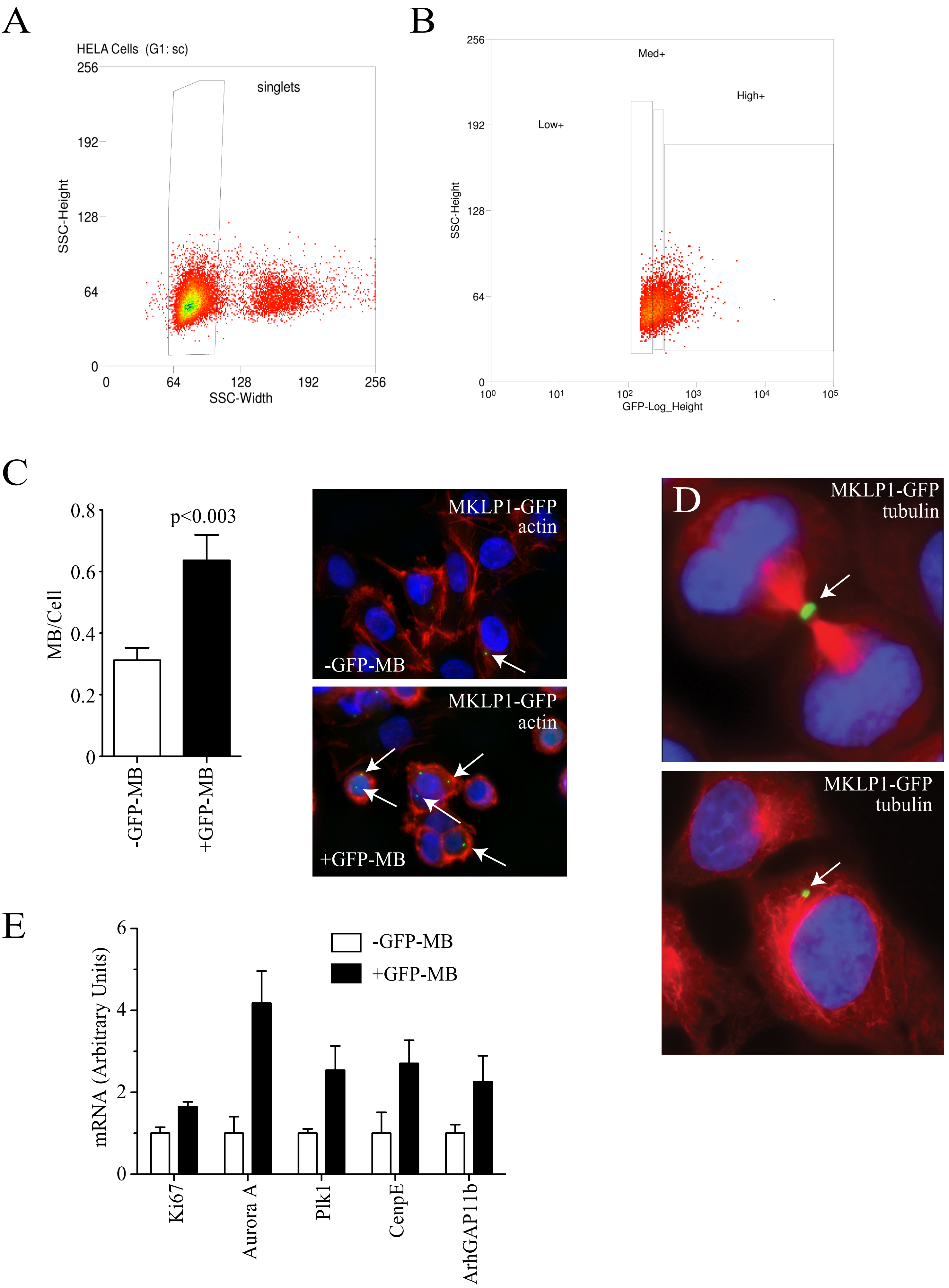

Supplementary Figure 2

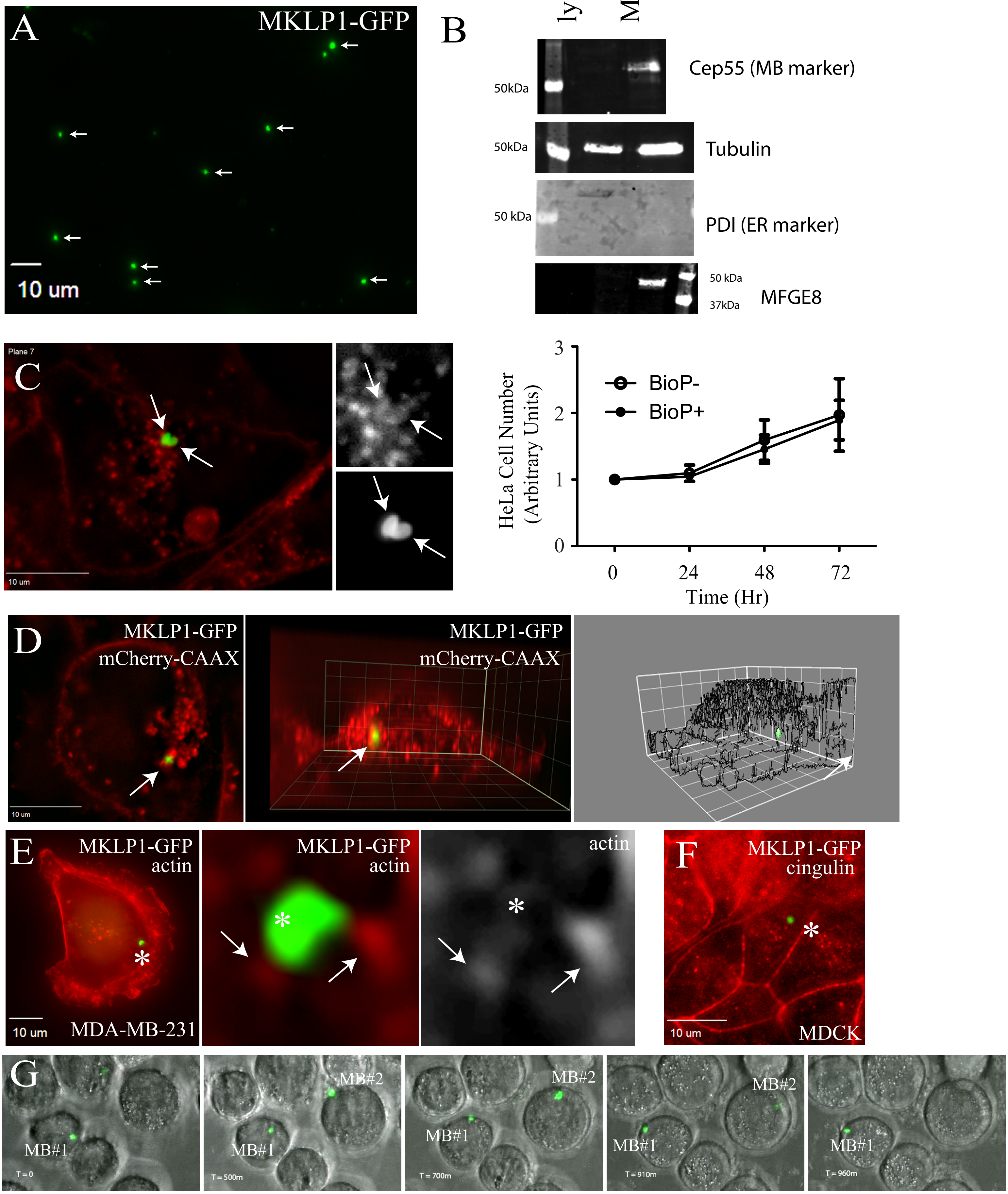

Supplementary Figure 3

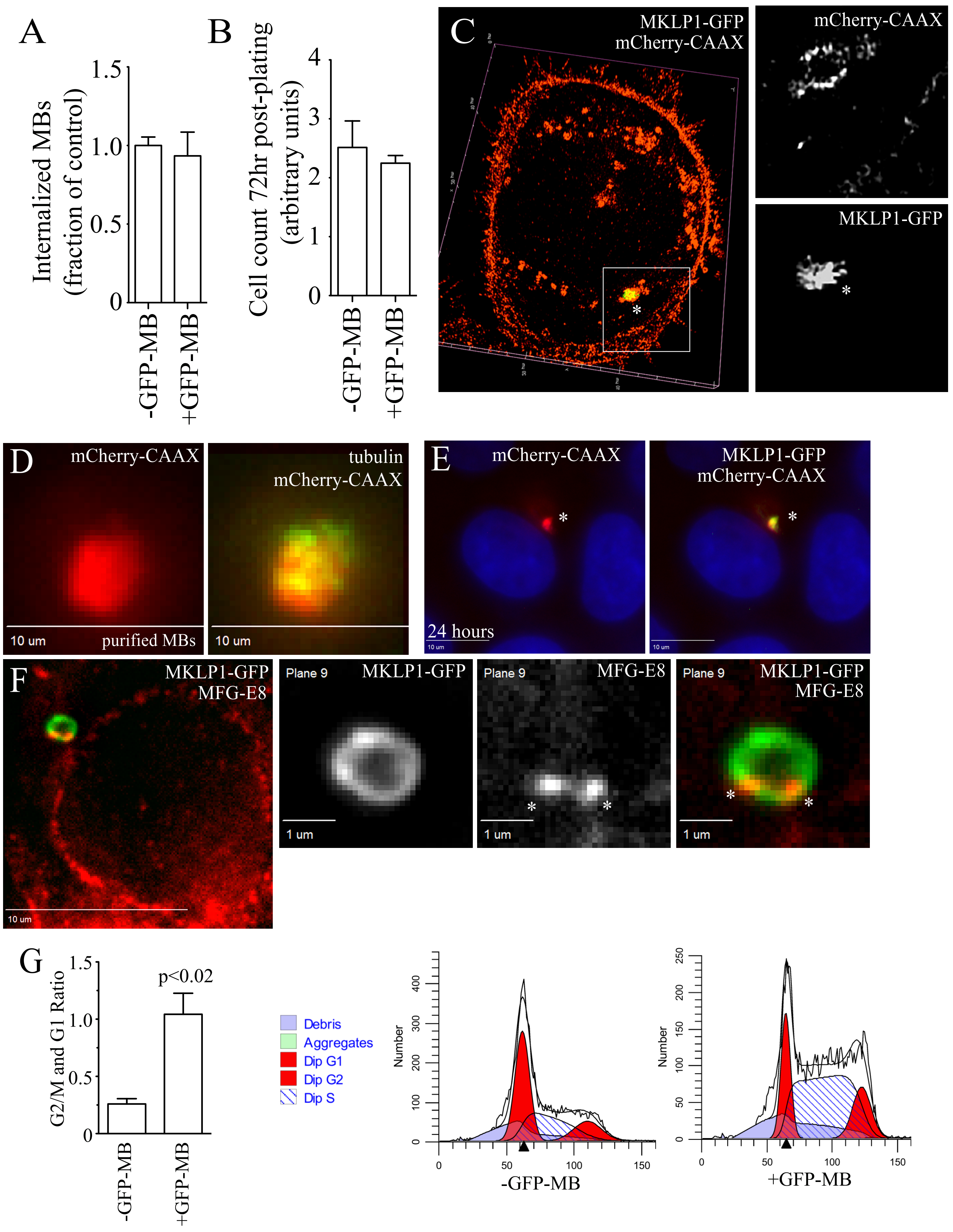

Supplementary Figure 4

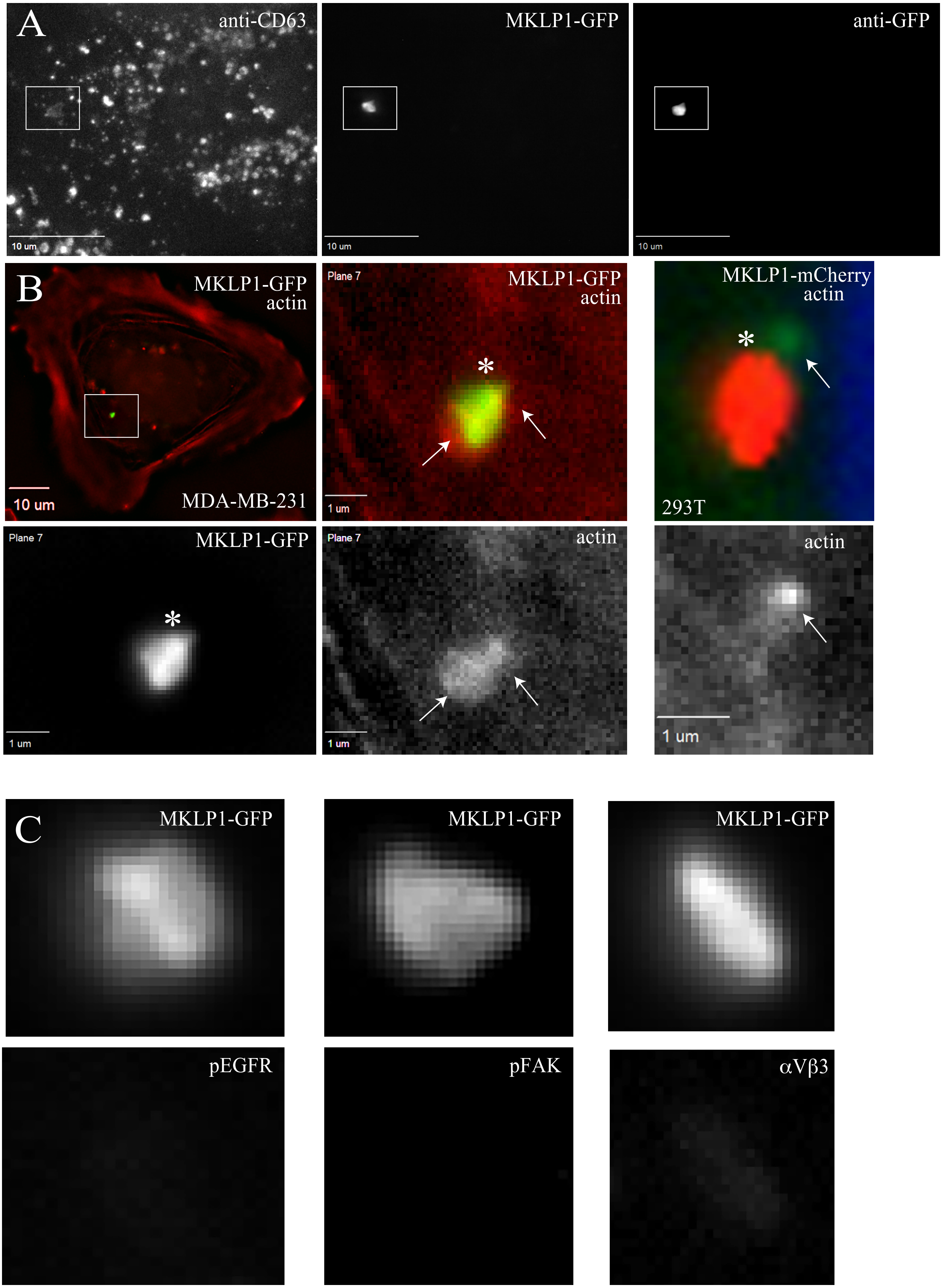

Supplementary Figure 5

A

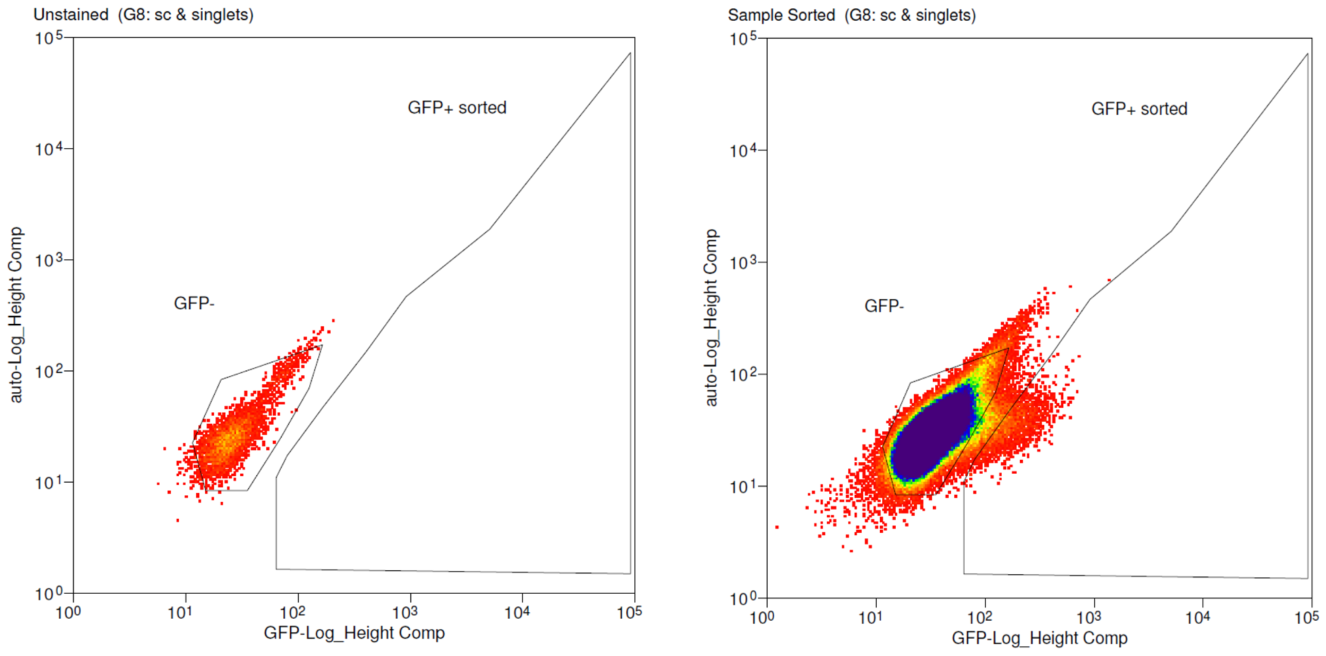


B

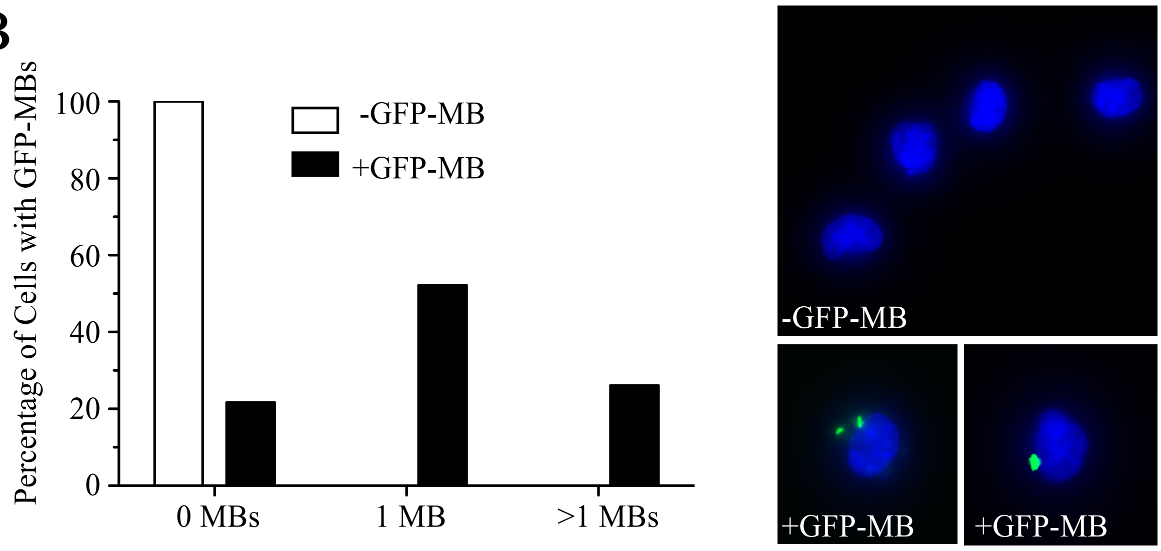

Supplementary Figure 6

Cep55

A.

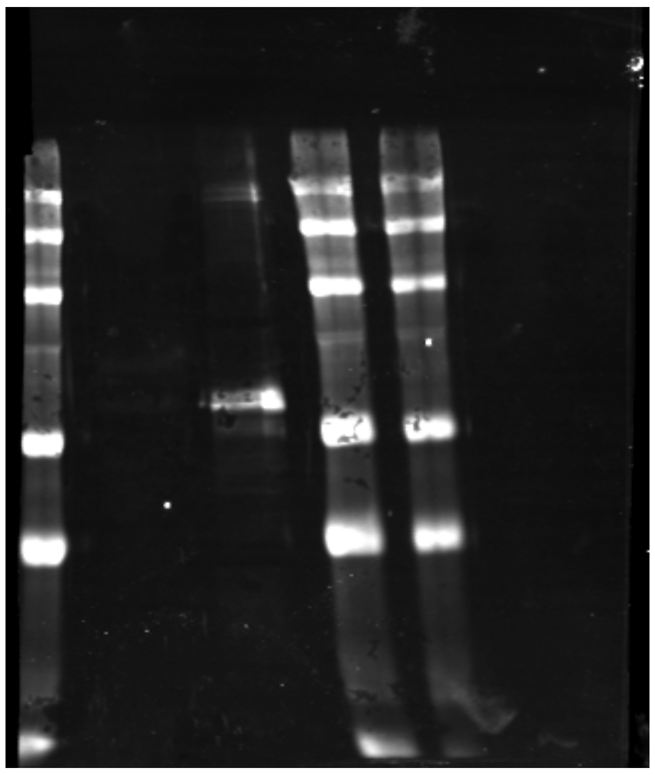

tubulin

B.

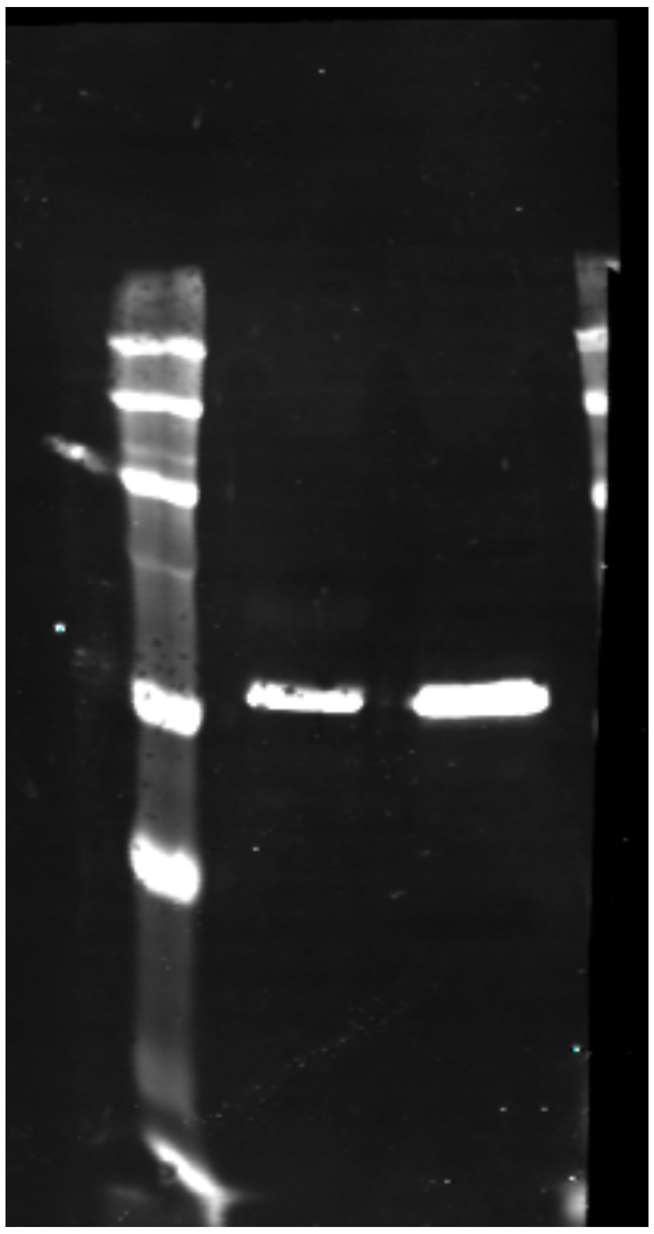

PDI

C.

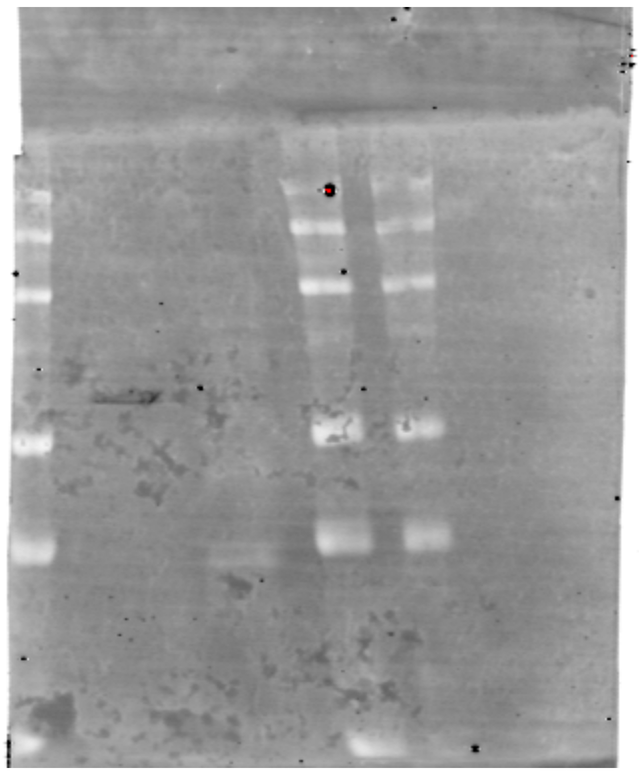

MFGE8

D.

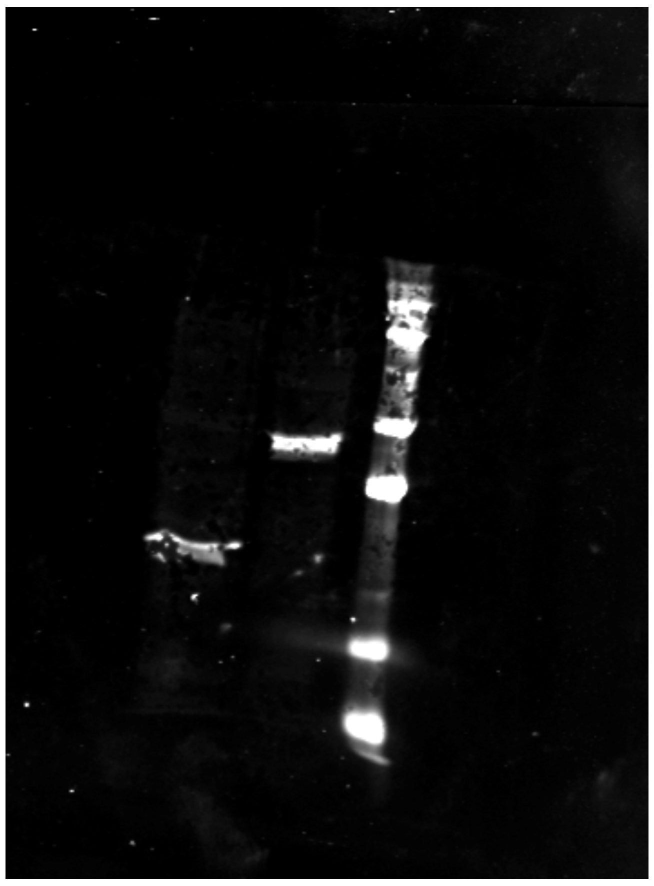

GST-LactC2

E.

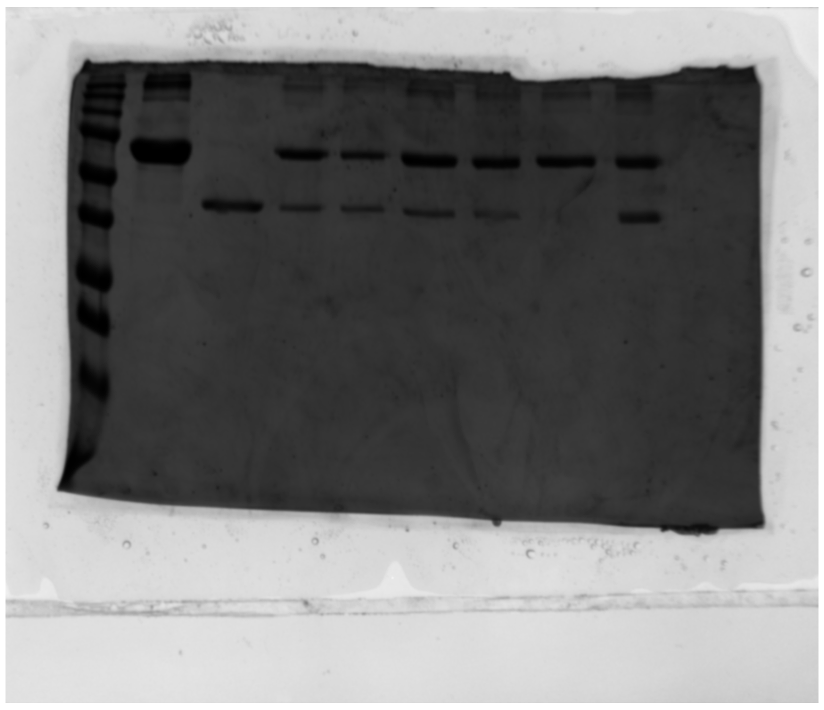

**Supplementary Figure 1. Accumulation of post-mitotic MB internalization stimulates cell proliferation**

(A-B) MKLP1-GFP expressing HeLa cells flow sorted for the enrichment of post-mitotic MBs. Panel A shows front-side scatter analysis. Panel B shown GFP fluorescence intensity analysis. Boxes mark the cells selected for sorting. Low GFP pool was designated as GFP- and High GFP pool was designated as GFP+.

(C) MKLP1-GFP expressing HeLa cells flow sorted for the enrichment of MBs were plated, fixed and analyzed for the presence or absence of MBs by fluorescent microscopy. The data shown are the means and standard deviations from three independent experiments (Unpaired, two-tailed Student's t-test).

(D) HeLa cells stably expressing MKLP1-GFP were fixed and stained with anti-acetylated tubulin antibodies. Arrow point to mitotic (top image) and post-mitotic (bottom image) MBs.

(E) MKLP1-GFP expressing HeLa cells flow sorted for the enrichment of MBs were analyzed for the levels of Ki67, Aurora A, Plk1, CenpE and ArhGAP 11b mRNAs by qPCR. The data shown are the means and standard deviations from four independent experiments (Unpaired, two-tailed Student's t-test)

**Supplementary Figure 2. Purification of GFP-labeled extracellular post-mitotic MBs**

(A) Image of purified GFP-MBs in suspension. Arrows point to individual MBs.

(B) Western blot analysis of Cep55 (MB marker), tubulin, PDI (ER marker) and MFG-E8 (PS and integrin binding protein) levels in purified GFP-MBs.

(C) HeLa cells expressing mCherry-CAAX were incubated with *Escherichia Coli* BioParticles (Alexa488-conjugated; ThermoFisher). Cells were then washed and flow-sorted for Alexa488 fluorescence. Sorted cells were either imaged (left images) or tested for their ability to proliferate (right bar graph). To ensure that BioParticles are situated inside the cell, Z-stack of 40 images was taken (step size 200 nm). Left panel shows single image taken from the middle of the Z-stack. Arrows point to internalized BioParticles. Data shown in graph represent means and standard deviations of three independent experiments (Unpaired, two-tailed Student's t-test).

(D) HeLa cells expressing mCherry-CAAX were incubated with GFP-MBs. Cells were then washed and incubated for another 24 hours before fixation. To ensure that MB is situated inside the cell, Z-stack of 40 images was taken (step size 200 nm). Left panel shows single images taken from the middle of the Z-stack. Middle panel shows 3D rendering of the image using all Z-stack. Panel on right shows surface 3D rendering derived from entire stack. Arrows point to the MB.

(E-F) MDA-MB-231 (E) and MDCK (F) cells were incubated with GFP-MBs (purified from HeLa cells). Cells were then washed and incubated for another 24 hours before fixation and staining with Alexa568-phalloidin (E) or anti-cingulin antibodies (tight junction marker; F). To ensure that MB is situated inside the cell, Z-stack of 40 images was taken (step size 200 nm). Images shown are single images from the middle of the Z-stack containing the MB. Arrows in E point to MB-associated actin. Asterisks in E and F mark MBs.

(G) HeLa cells were incubated with purified GFP-MBs for 3 hours. Cells were then washed and imaged by time-lapse microscopy for 16 hours. At every time-point (20 min) z-stack of 10 μm (1 μm step) was

taken to ensure that GFP-MBs are always in-focus. Shown images are z-projections of selected individual time point.

**Supplementary Figure 3. The effect of MBs in regulating cell proliferation**

(A-B) HeLa cells were incubated with purified GFP-MBs. Cells were then washed and flow sorted to isolate cells with (+GFP-MB) or without (-GFP-MB) internalized MBs. Both cell populations were then grown for 7 days and re-plated to measure their proliferation rates (B) and ability to internalize MBs (A). Data shown in graph represent means and standard deviations of three independent experiments.

(C) HeLa cells stably expressing mCherry-CAAX were incubated with GFP-MBs for 3 hours. Cells were then washed and incubated for another 24 hours to allow MB internalization and then imaged using SIM super-resolution microscope. Asterisk marks internalized MB. Box marks the region shown in higher magnification images on the right.

(D) Midbodies were isolated from HeLa mCherry-CAAX cells, adhered to a poly-l-lysine coated coverslip, then stained for acetylated tubulin.

(E) Midbodies were isolated from HeLa cells expressing mCherry-CAAX and MKLP1-GFP. Midbodies were then incubated with normal HeLa cells and stained with Hoecsht 33342.

(F) HeLa cells were incubated with GFP-MBs for 1 hour to allow GFP-MB to cell surface. Cells were then washed, fixed and stained with anti-MFG-E8 antibodies. Asterisks mark plasma membrane and MB contact sites.

(G) HeLa cells were flow-sorted in GFP-MB positive and negative populations. The number of cells in G1 or G2/M was then analyzed by flow cytometry based on their DNA content. The data shown are the means and standard deviations of three independent experiments (Unpaired, two-tailed Student's t-test).

**Supplementary Figure 4. The association of MBsomes with CD63, actin, integrins and EGFR**

(A) HeLa cells were incubated with purified GFP-MBs. Cells were then washed and incubated for another 24 hours before fixation and staining with anti-CD63 and anti-GFP. To ensure that MB is situated inside the cell rather than on cell surfaces, Z-stack of 40 images was taken (step size 200 nm). Images shown are single images from the middle of the Z-stack containing the MB. Boxes mark the internalized post-mitotic MB.

(B) MDA-MB-231 (left panels) or 293T (right panels) cells were incubated with purified GFP-MBs or mCherry-MBs. Cells were then washed and incubated for another 24 hours before fixation and staining with Alexa568-phalloidin or phalloidin iFluor 488. To ensure that MB is situated inside the cell rather than on cell surfaces, Z-stack of 40 images was taken (step size 200 nm). Images shown are single images from the middle of the Z-stack containing the MB. Arrows point to MB-associated actin. Asterisks the midbodies.

(C) Purified GFP-MBs were plated on poly-L-lysine coverslip and stained with anti-pEGFR, anti-pFAK and anti-$\alpha V\beta 3$ antibodies.

**Supplementary Figure 5. Flow sorting of HeLa cells with internalized post-mitotic GFP-MBs**

(A) HeLa cells were incubated with purified GFP-MBs and then flow sorted for cells with and without internalized post-abscission MBs. Panel A shows un-fed cells while Panel B shows cells that were incubated with GFP-MBs. Boxes mark the cells selected for sorting.

(B) HeLa cells flow sorted for the presence of internalized GFP-MB were plated, fixed and analyzed for the presence of GFP-MBs.

**Supplementary Figure 6. Uncropped protein blots.**

(A-D). Uncropped Western Blots corresponding to the blots shown in Supplementary Figure 2B.

(E). Uncropped Coomassie-stained blot corresponding to the blot shown in Figure 5A.
